# Supplementary material for: The interactome of intact mitochondria by cross-linking mass spectrometry provides evidence for coexisting respiratory supercomplexes
Source: Mol Cell Proteomics. 2017 Dec 8;17(2):216–32. doi: 10.1074/mcp.RA117.000470 (PMC5795388; doi:10.1074/mcp.RA117.000470)
Supplement: Supplemental Data [file supp_RA117.000470_133922_0_supp_23978_fzffwf.zip › spectra_annotation/mito_spectra_annotation/325-1-8-1-22-1.pdf]

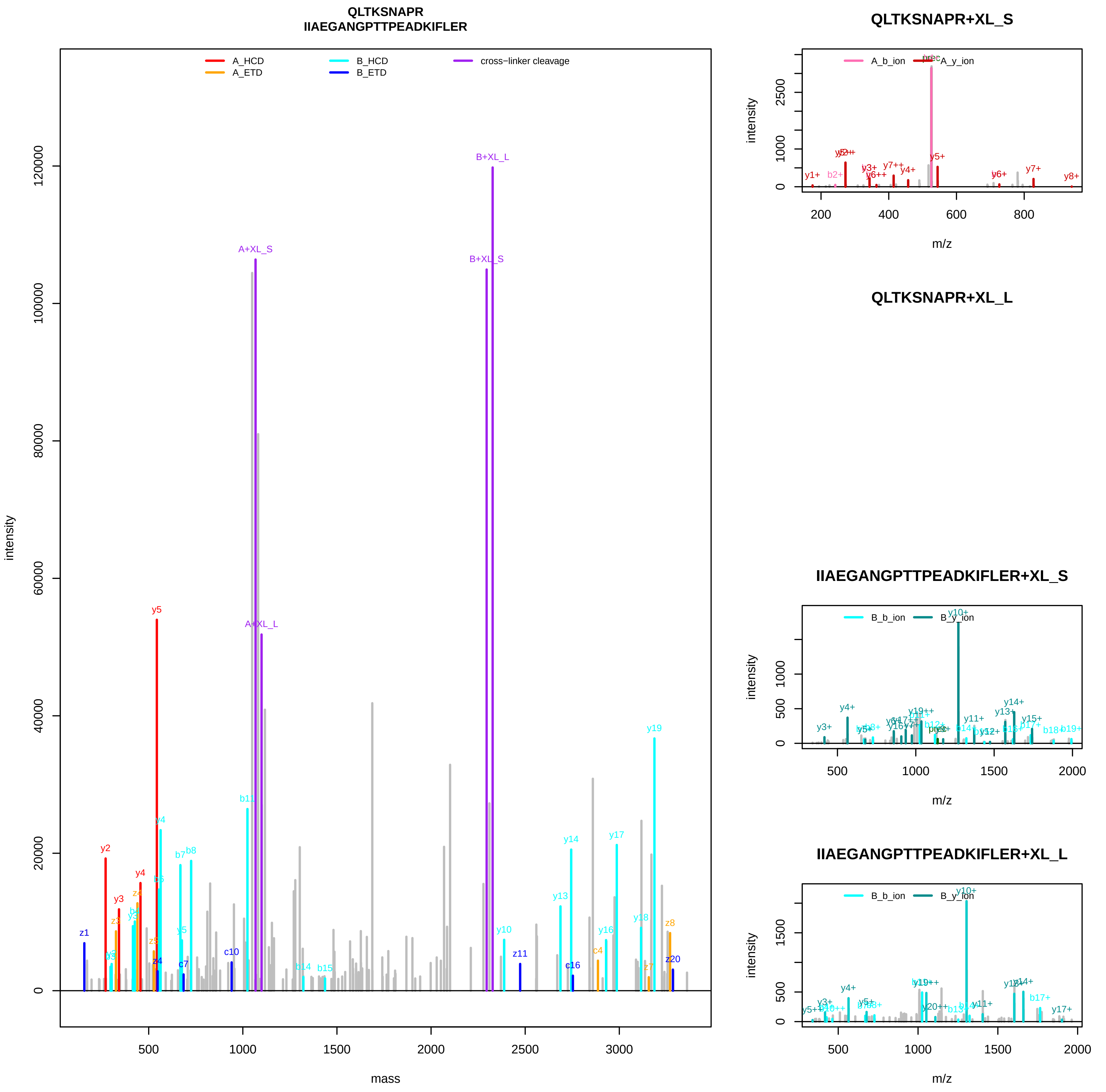

QLTKSNAPR
IIAEGANGPTTPEADKIFLER
A_HCD
A_ETD
B_HCD
B_ETD
cross−linker cleavage
intensity
mass
B+XL_L
A+XL_S
B+XL_S
A+XL_L
y5
y2
y3
y4
z1
b11
y4
b7
b8
c10
c7
b14
b15
y10
z11
c16
y13
y14
y16
y17
y18
y19
z8
z20
c4
QLTKSNAPR+XL_S
A_b_ion
A_y_ion
m/z
QLTKSNAPR+XL_L
IIAEGANGPTTPEADKIFLER+XL_S
B_b_ion
B_y_ion
IIAEGANGPTTPEADKIFLER+XL_L
